# Curari-Woorari—A Cure for Hydrophobia

**Published:** 1878-01

**Authors:** 


CURARI-WOORARI—A CURE FOR HYDROPHOBIA.—At Munster, Westphalia, Germany, a girl was bitten by a mad dog the 23d of July. On the 16th of October following, hydrophobia manifested itself, and morphia and chloroform were futile in allaying the spasms. Dr. Offenburg then injected three centigrammes of curari-woorari, and repeated it five times, though not always in equal quantity, for four hours and a half. The convulsions began to diminish after the second injection, and soon disappeared. Paralysis of the chest was prevented by artificial respiration, and in two weeks the girl was well again. The remedy is a terrible one, but the disease is still more so, but the success of any remedy will be hailed with the greatest satisfaction by humanity.

